# Supplementary figures and images for: Mechanism of endothelial nitric oxide synthase phosphorylation and activation by tentacle extract from the jellyfish Cyanea capillata
Source: PeerJ. 2017 Apr 11;5:e3172. doi: 10.7717/peerj.3172 (PMC5390764; doi:10.7717/peerj.3172)

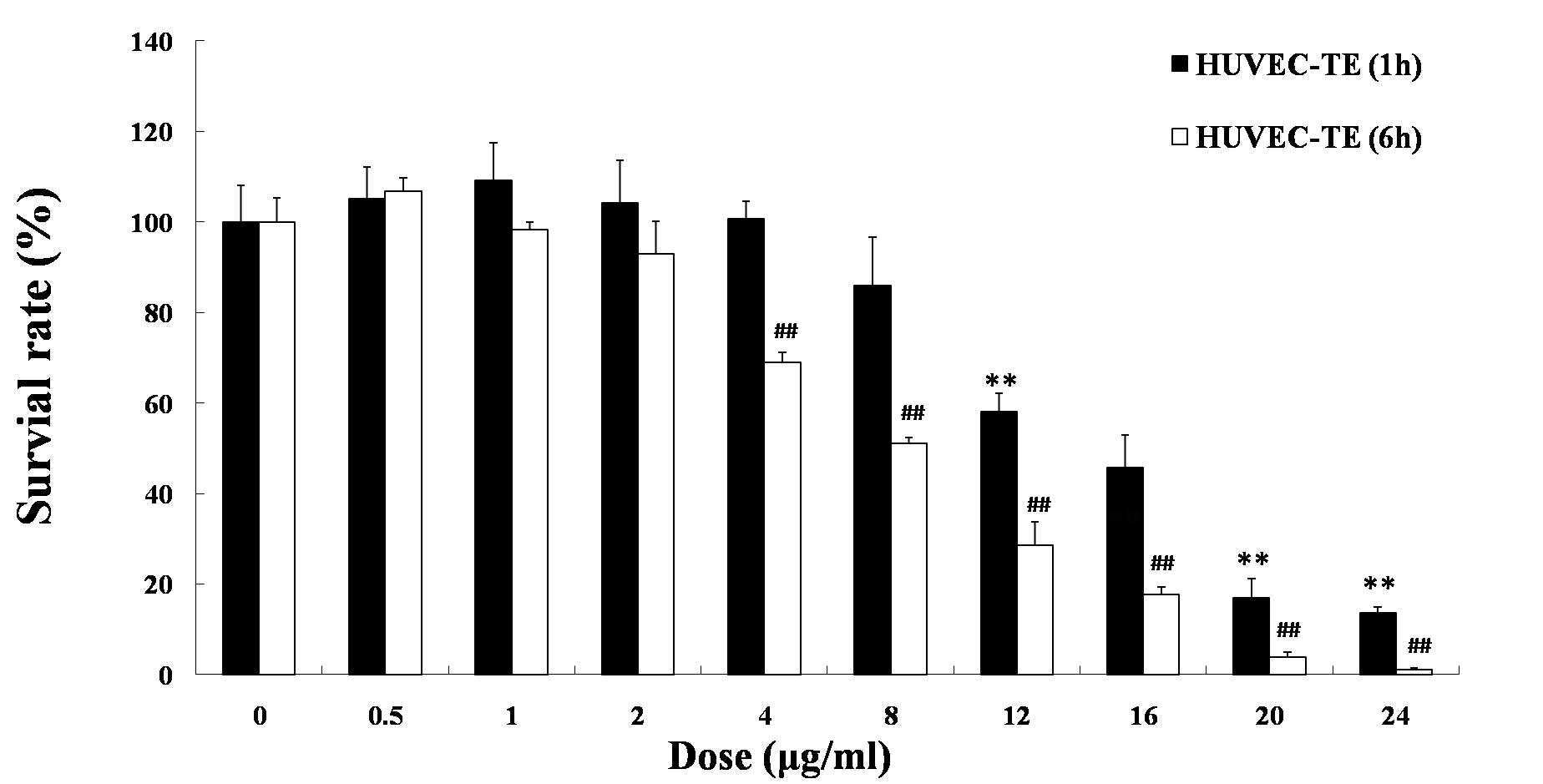

Supplement: Data S1 [file peerj-05-3172-s001.zip › raw data/Fig.1/Fig.1.tif]

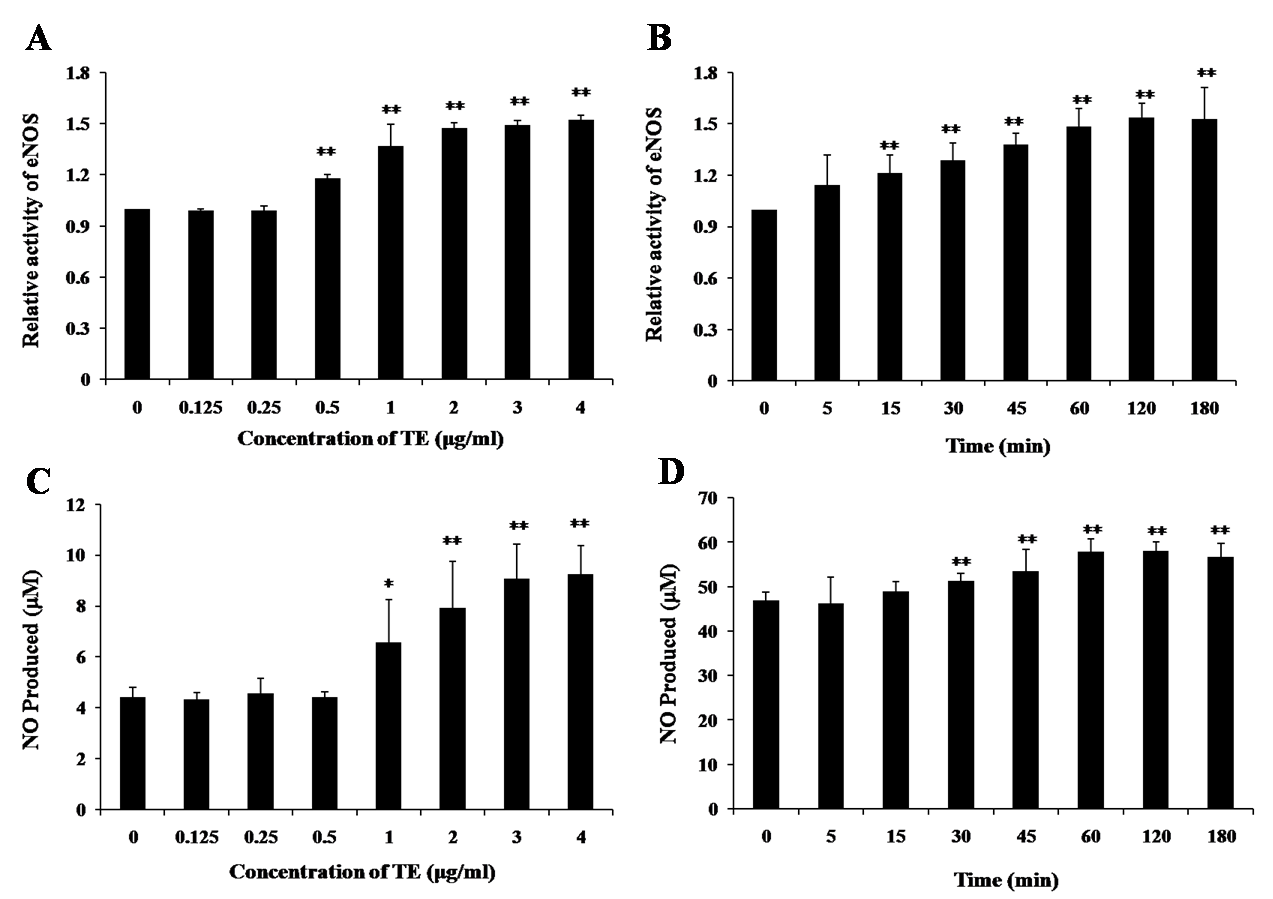

Supplement: Data S1 [file peerj-05-3172-s001.zip › raw data/Fig.2/Fig.2.tif]

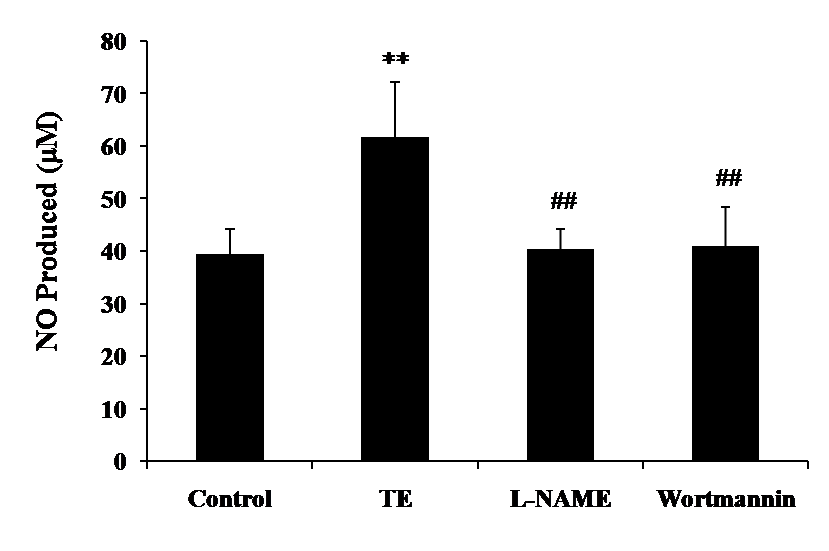

Supplement: Data S1 [file peerj-05-3172-s001.zip › raw data/Fig.4/Fig.4.tif]

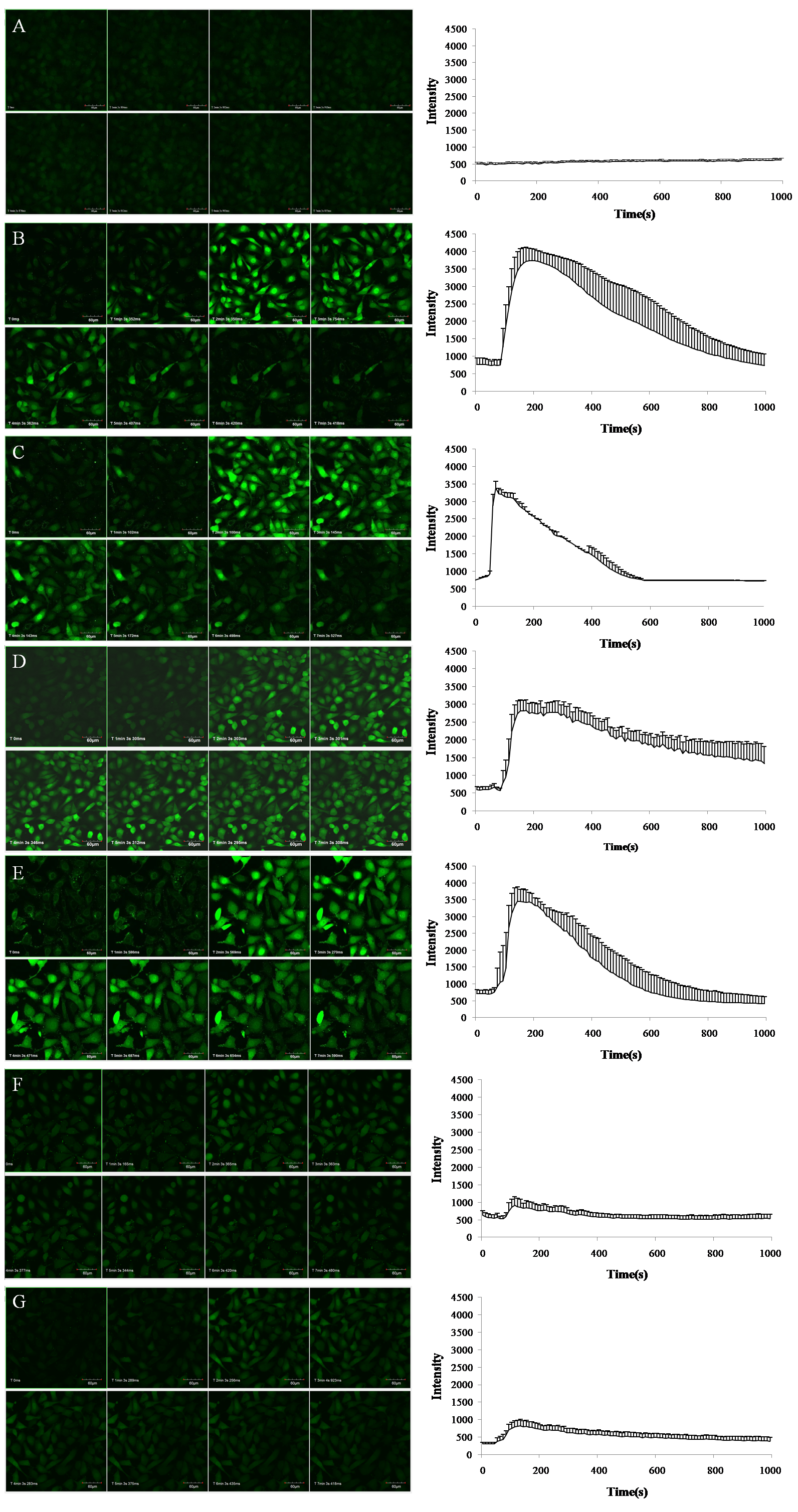

Supplement: Data S1 [file peerj-05-3172-s001.zip › raw data/Fig.5/Fig.5.tif]

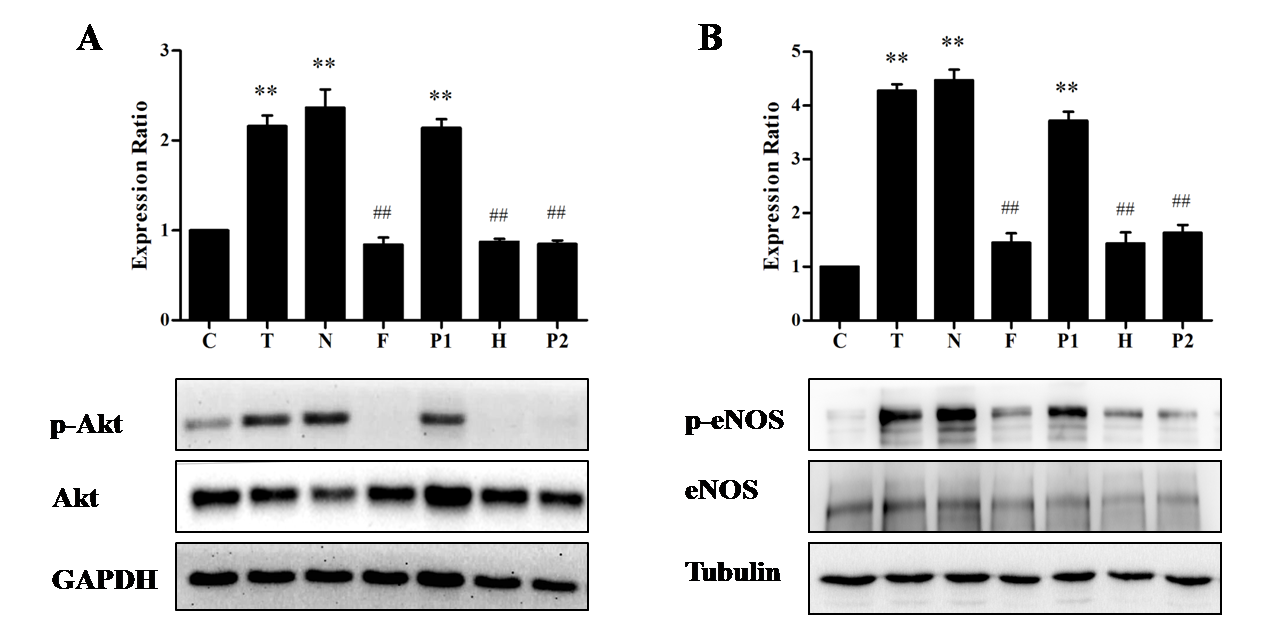

Supplement: Data S1 [file peerj-05-3172-s001.zip › raw data/Fig.6/Fig.6.tif]

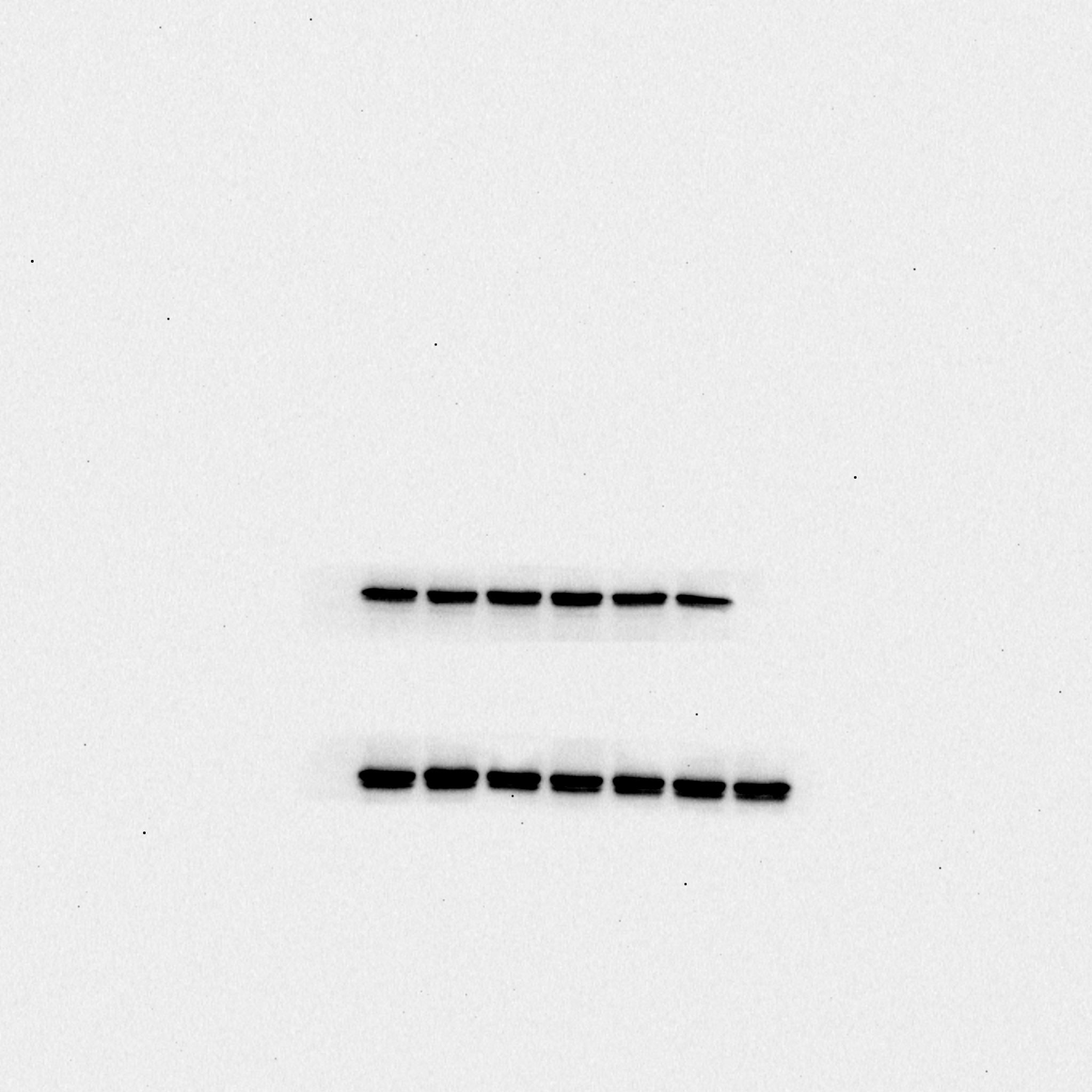

Supplement: Supplemental Information 1 [file peerj-05-3172-s002.zip › Figures 3 and 6/Fig 3/Fig 3A. Akt.tif]

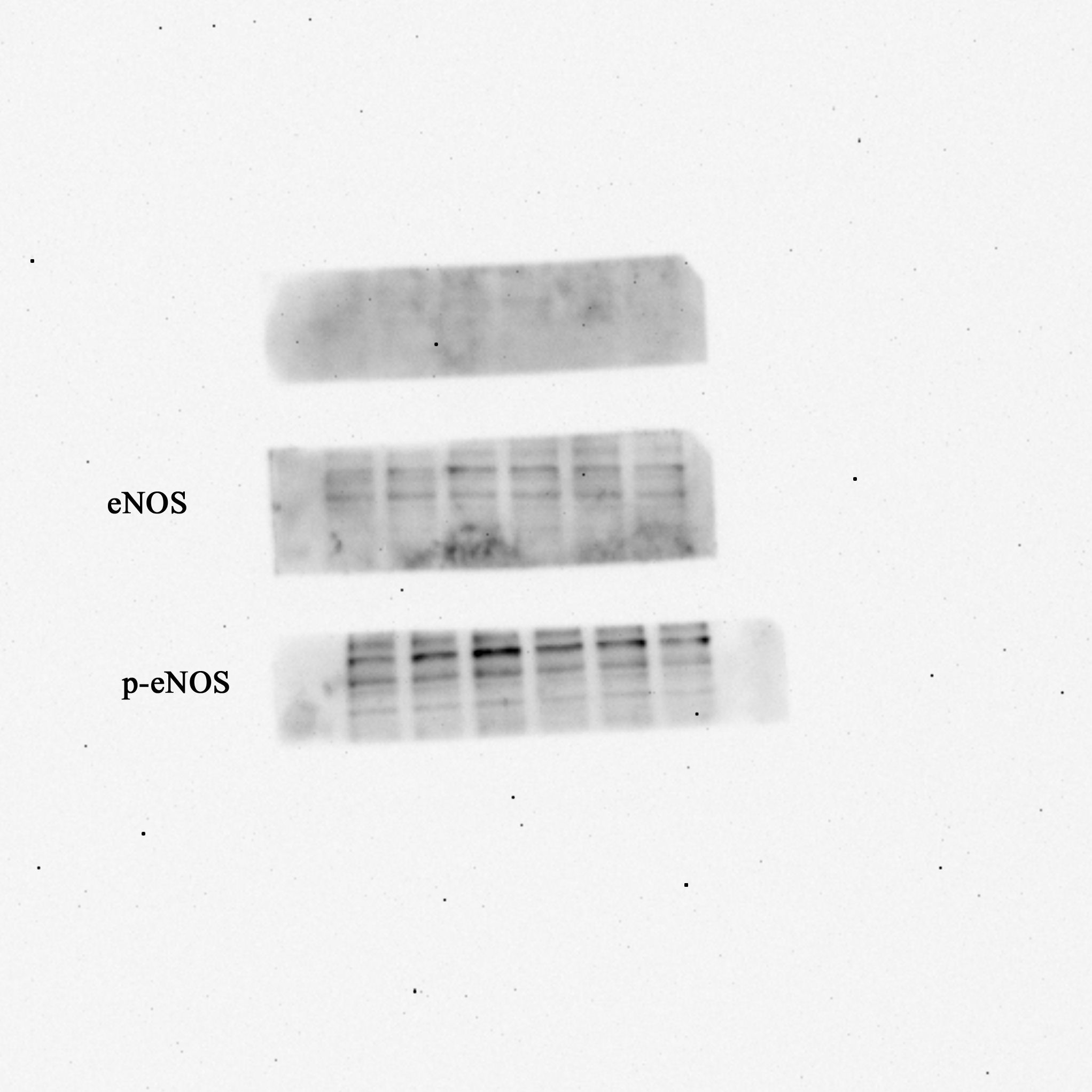

Supplement: Supplemental Information 1 [file peerj-05-3172-s002.zip › Figures 3 and 6/Fig 3/Fig 3A.eNOS(middle).tif]

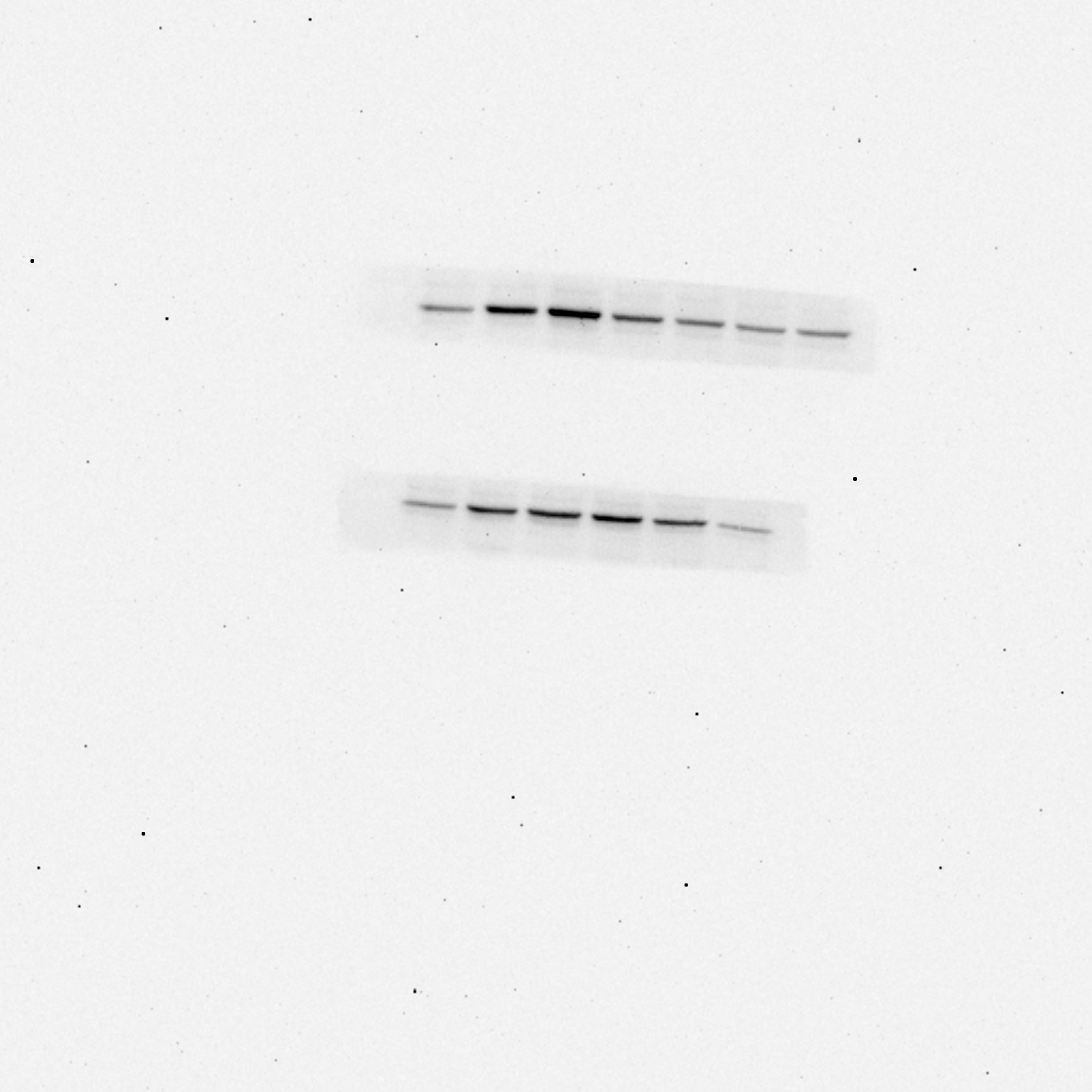

Supplement: Supplemental Information 1 [file peerj-05-3172-s002.zip › Figures 3 and 6/Fig 3/Fig 3A.P-Akt.tif]

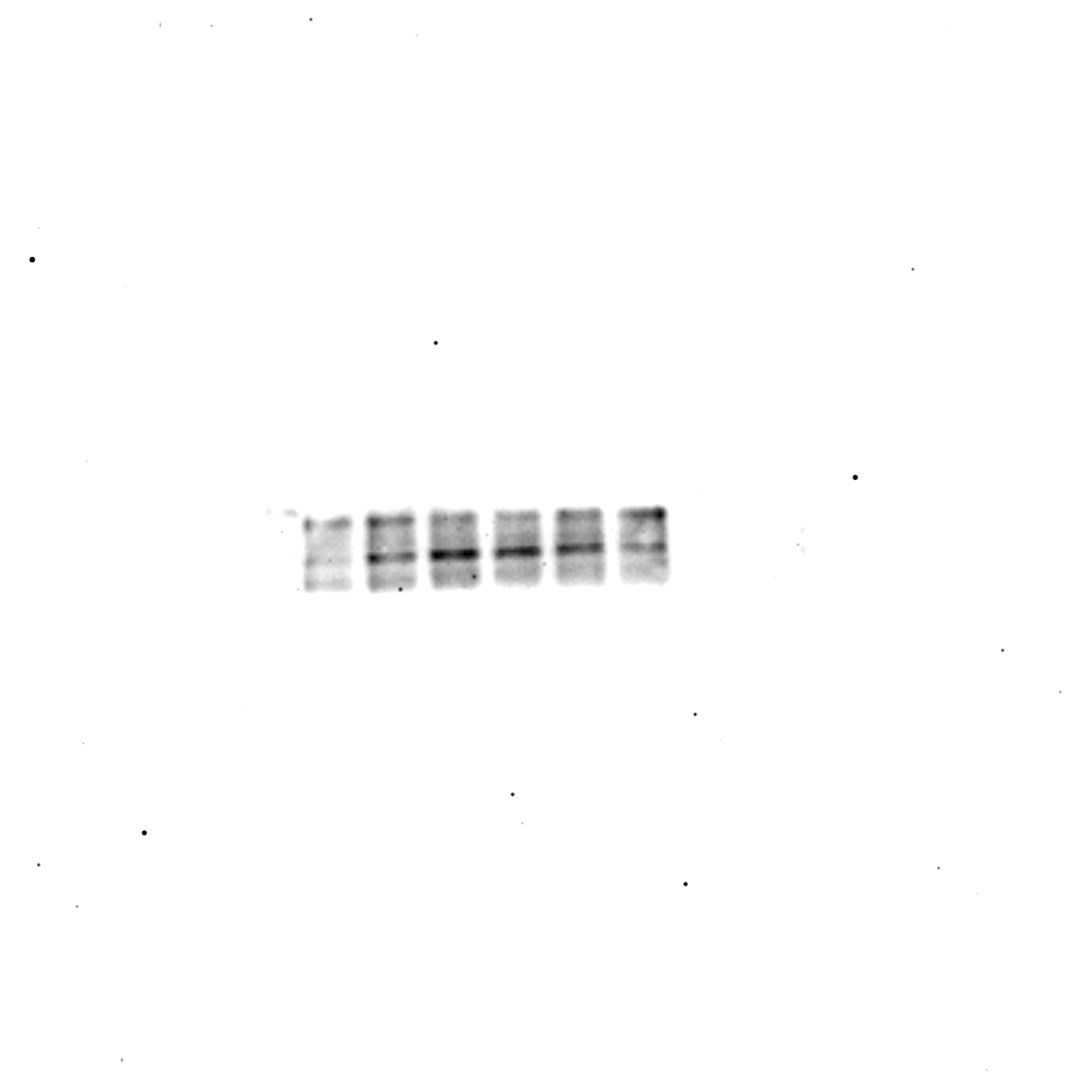

Supplement: Supplemental Information 1 [file peerj-05-3172-s002.zip › Figures 3 and 6/Fig 3/Fig 3A.p-eNOS.tif]

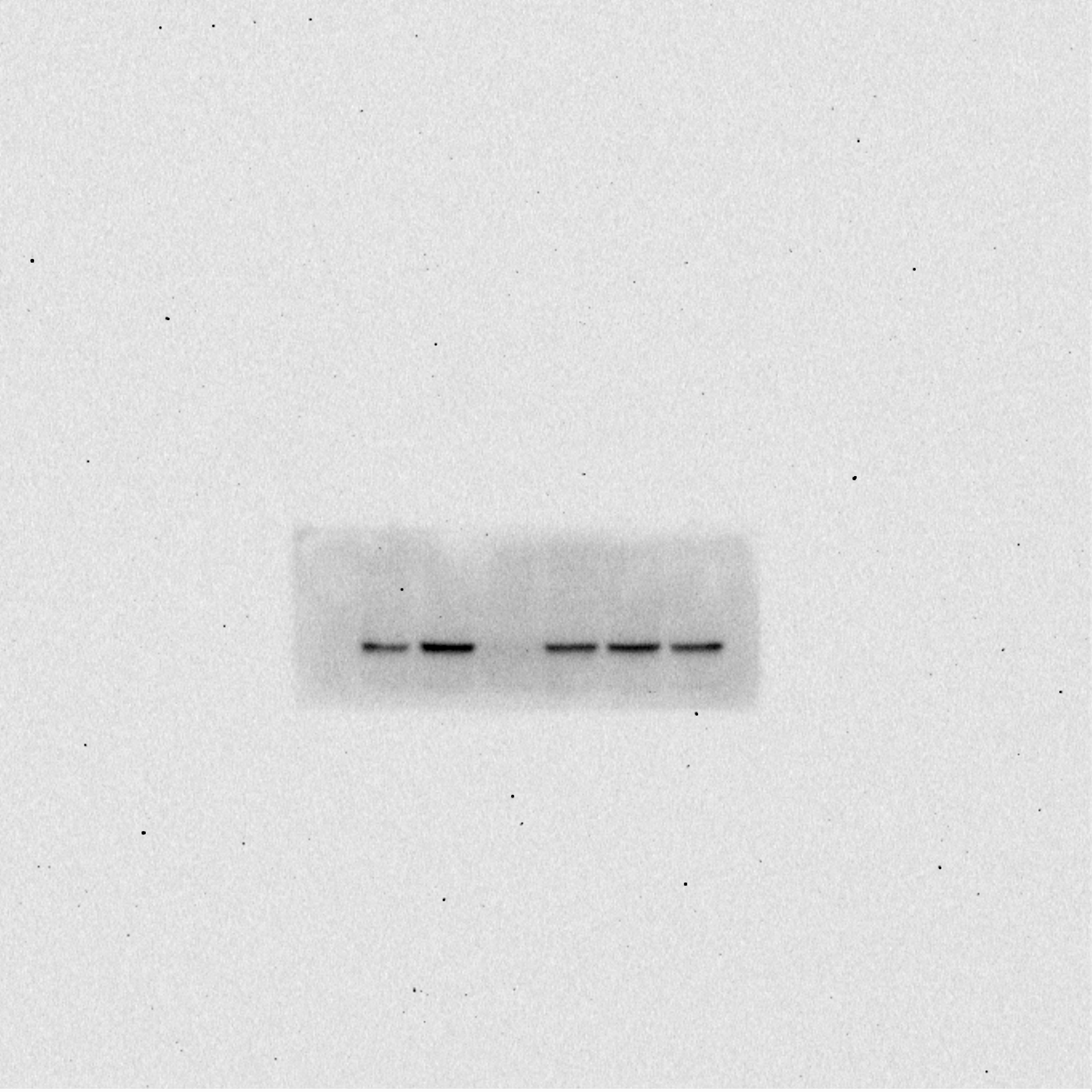

Supplement: Supplemental Information 1 [file peerj-05-3172-s002.zip › Figures 3 and 6/Fig 3/Fig 3B.inhibitor-P-AKT-.tif]

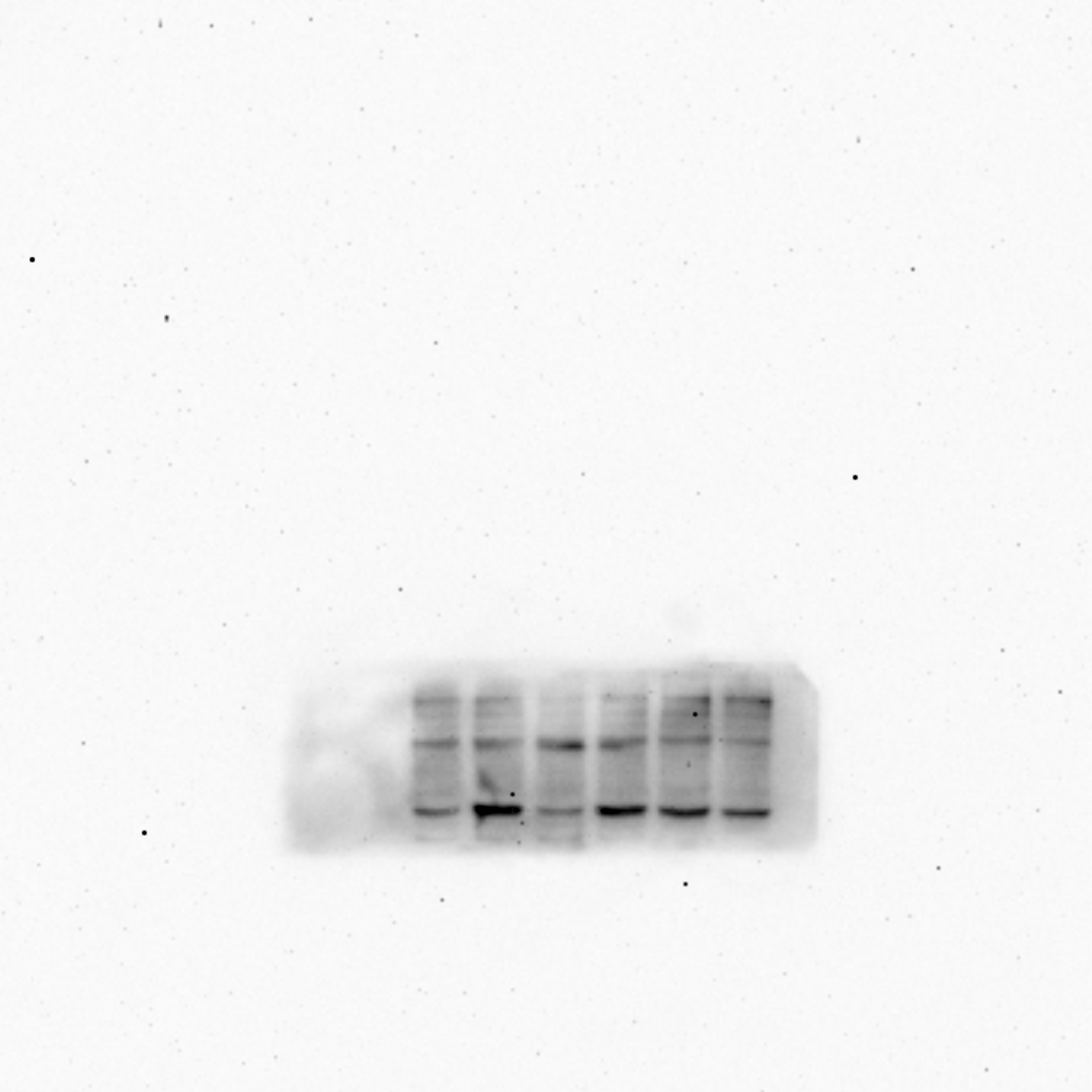

Supplement: Supplemental Information 1 [file peerj-05-3172-s002.zip › Figures 3 and 6/Fig 3/Fig 3B.inhibitor-P-eNOS-.tif]

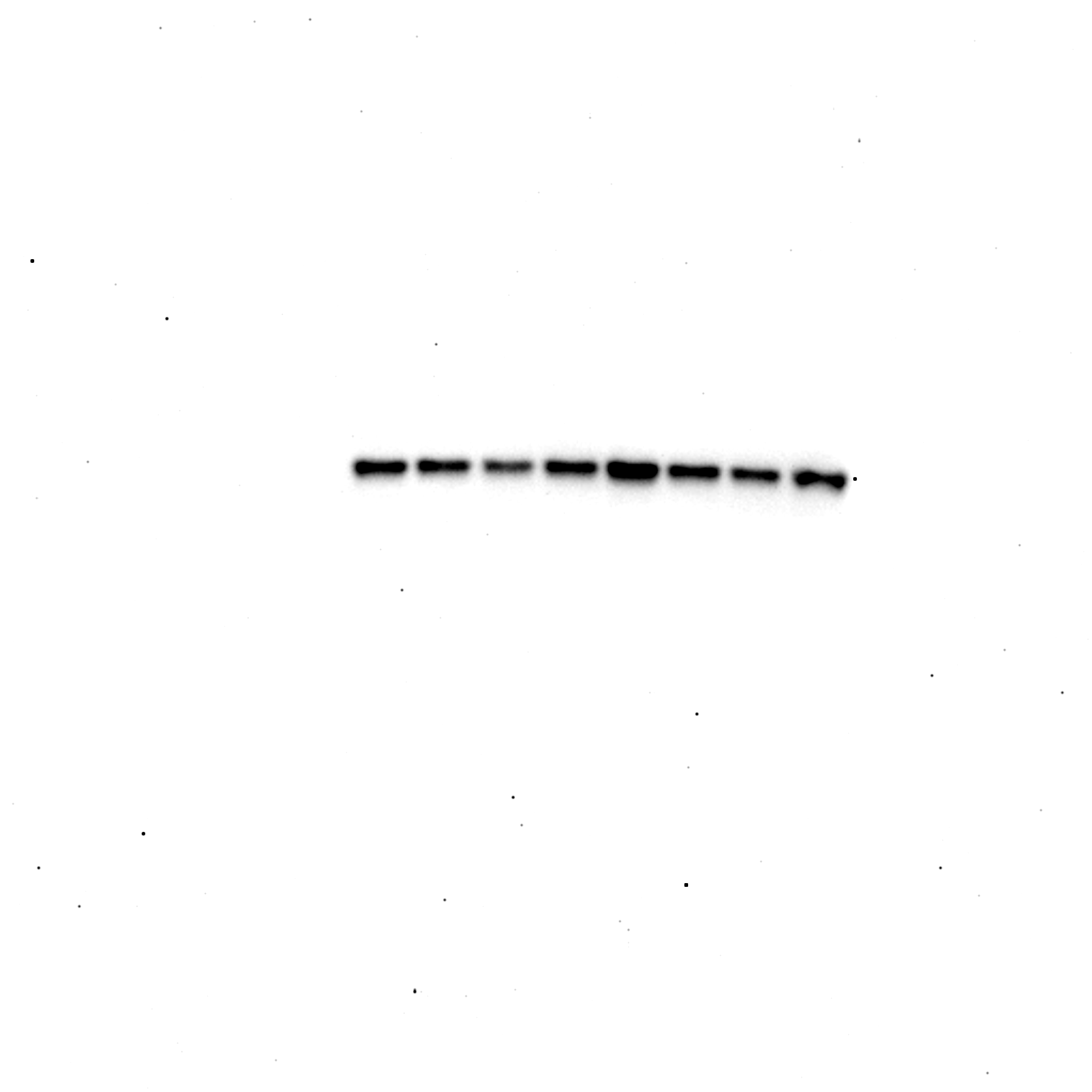

Supplement: Supplemental Information 1 [file peerj-05-3172-s002.zip › Figures 3 and 6/Fig 6/Fig6. AKT1-.tif]

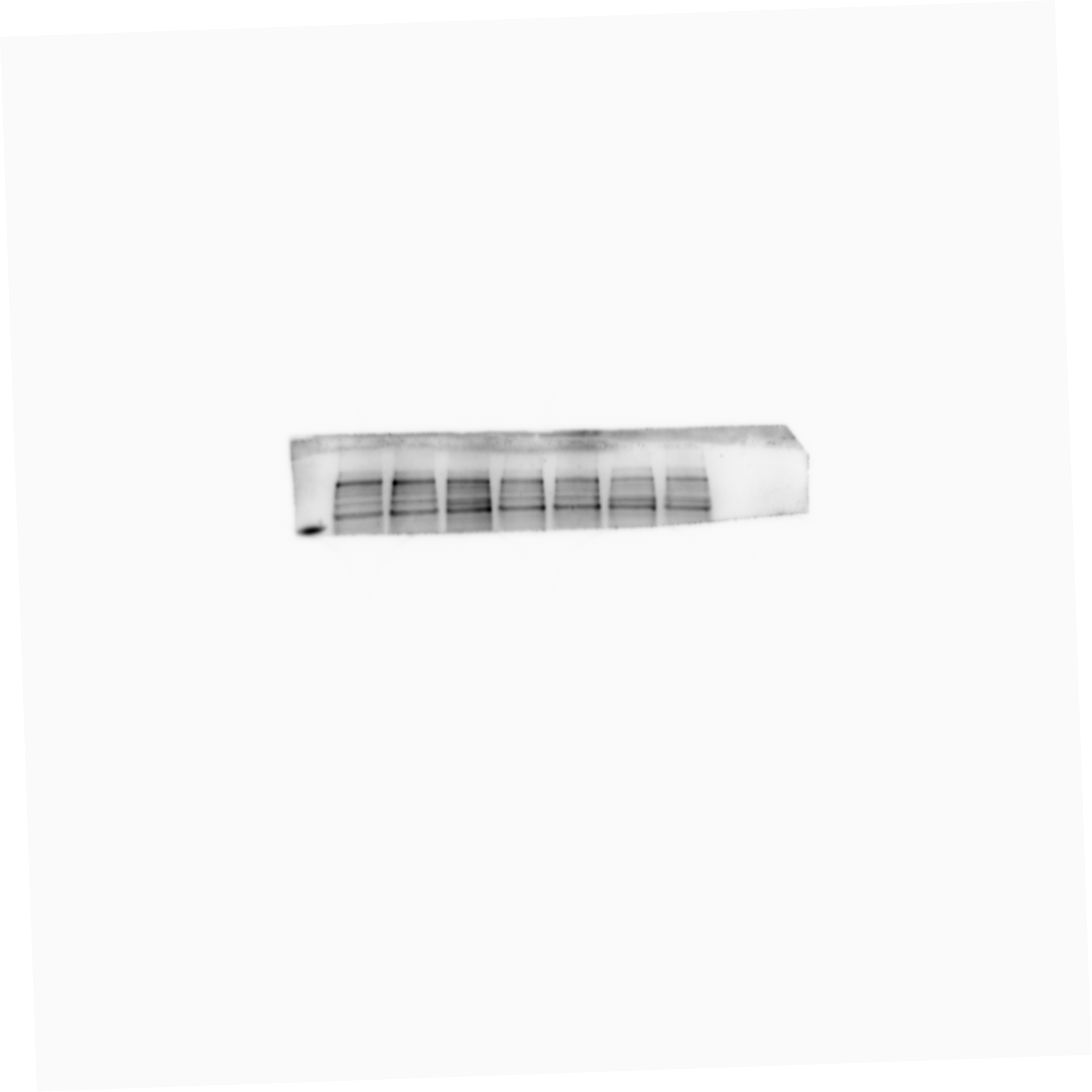

Supplement: Supplemental Information 1 [file peerj-05-3172-s002.zip › Figures 3 and 6/Fig 6/Fig6. eNOS.tif]

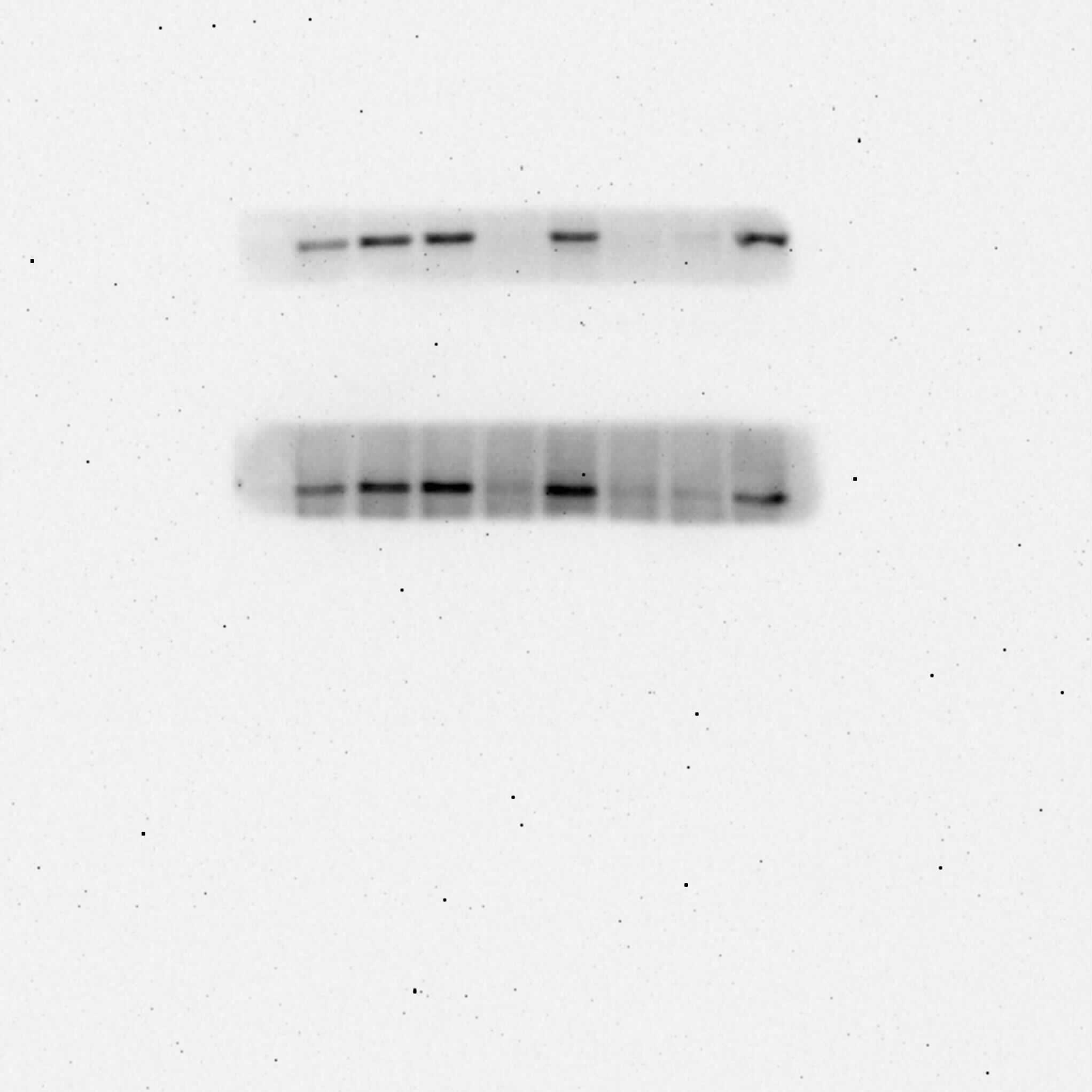

Supplement: Supplemental Information 1 [file peerj-05-3172-s002.zip › Figures 3 and 6/Fig 6/Fig6. P-AKT-.jpg]

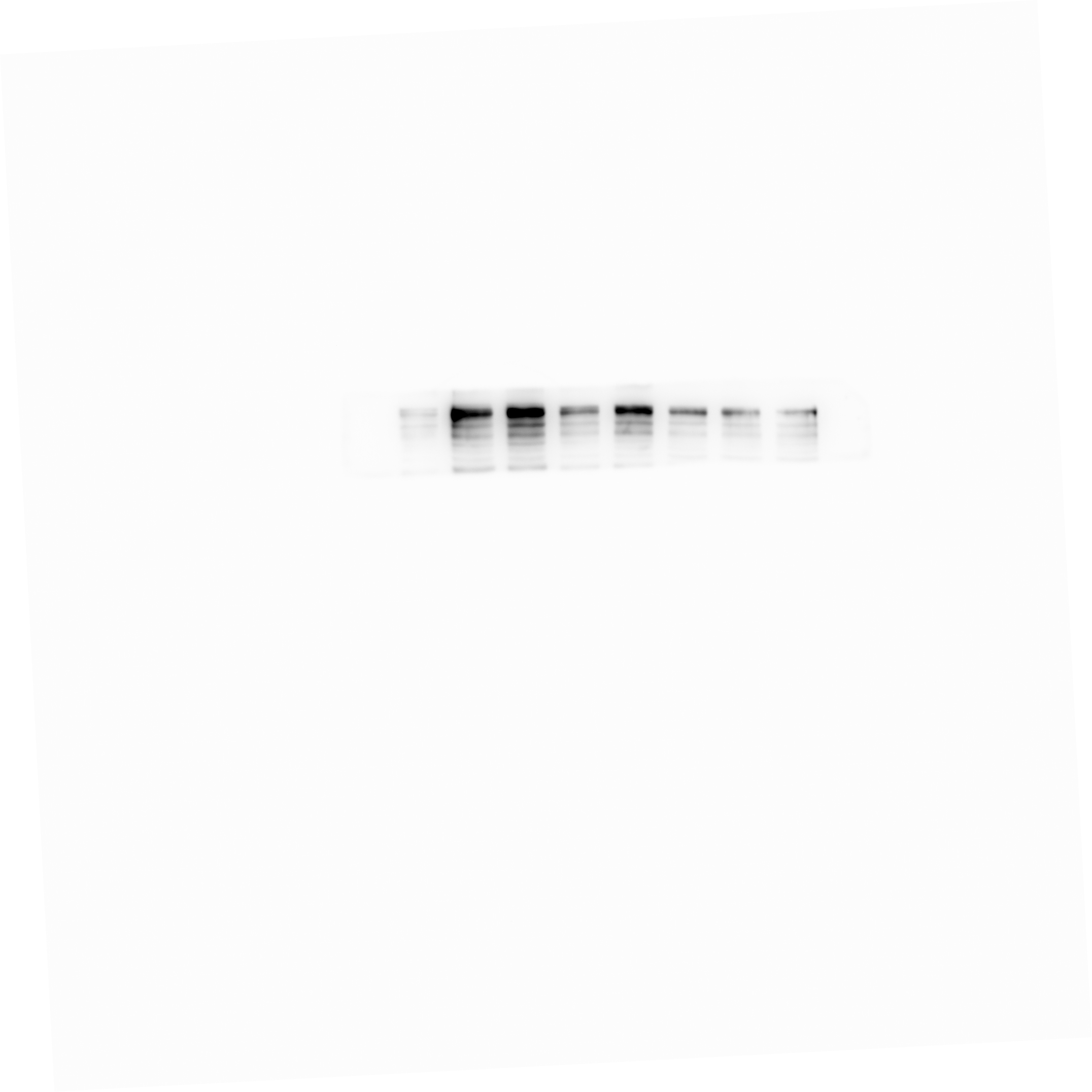

Supplement: Supplemental Information 1 [file peerj-05-3172-s002.zip › Figures 3 and 6/Fig 6/Fig6. p-eNOS.tif]

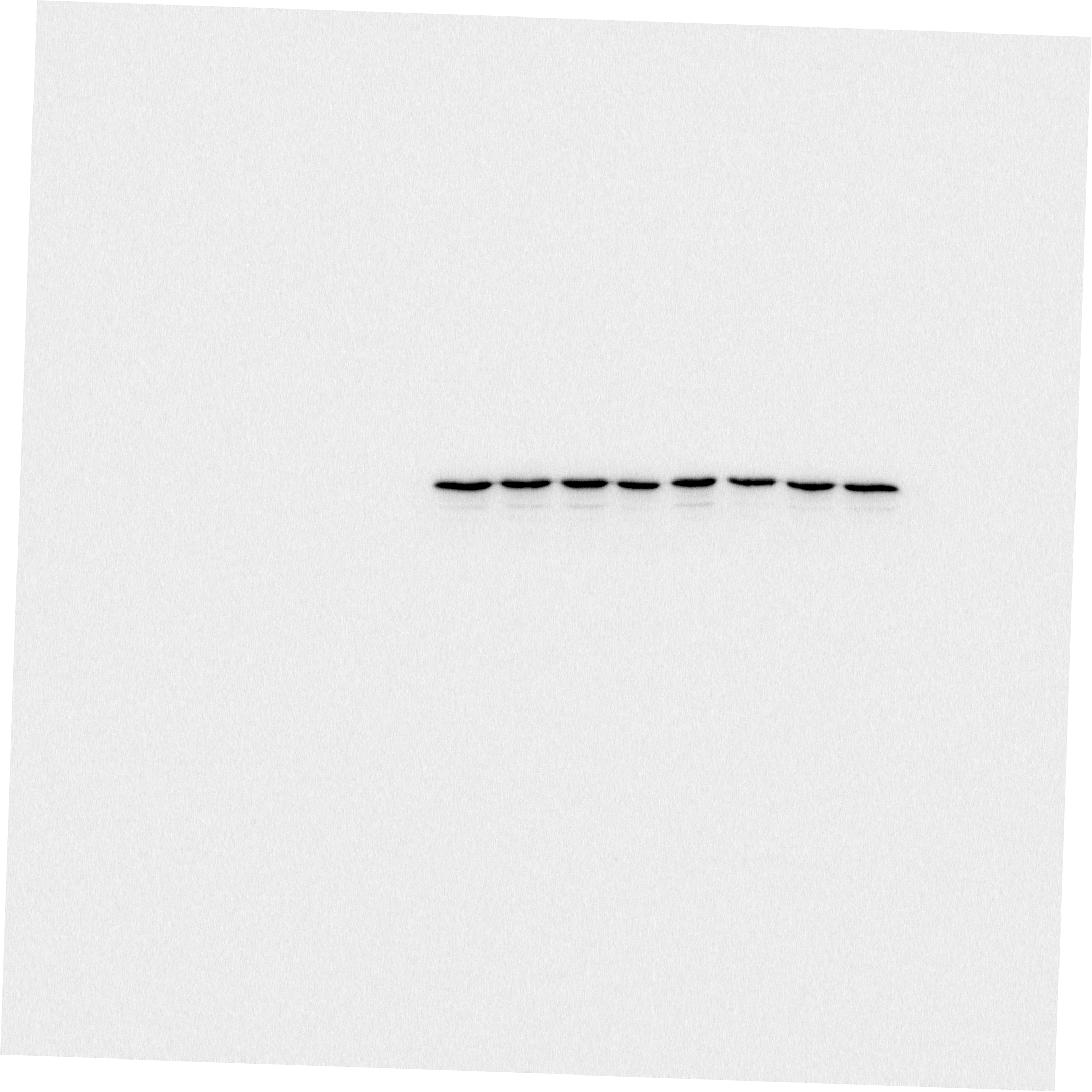

Supplement: Supplemental Information 1 [file peerj-05-3172-s002.zip › Figures 3 and 6/Fig 6/Fig6. Tubulin.tif]

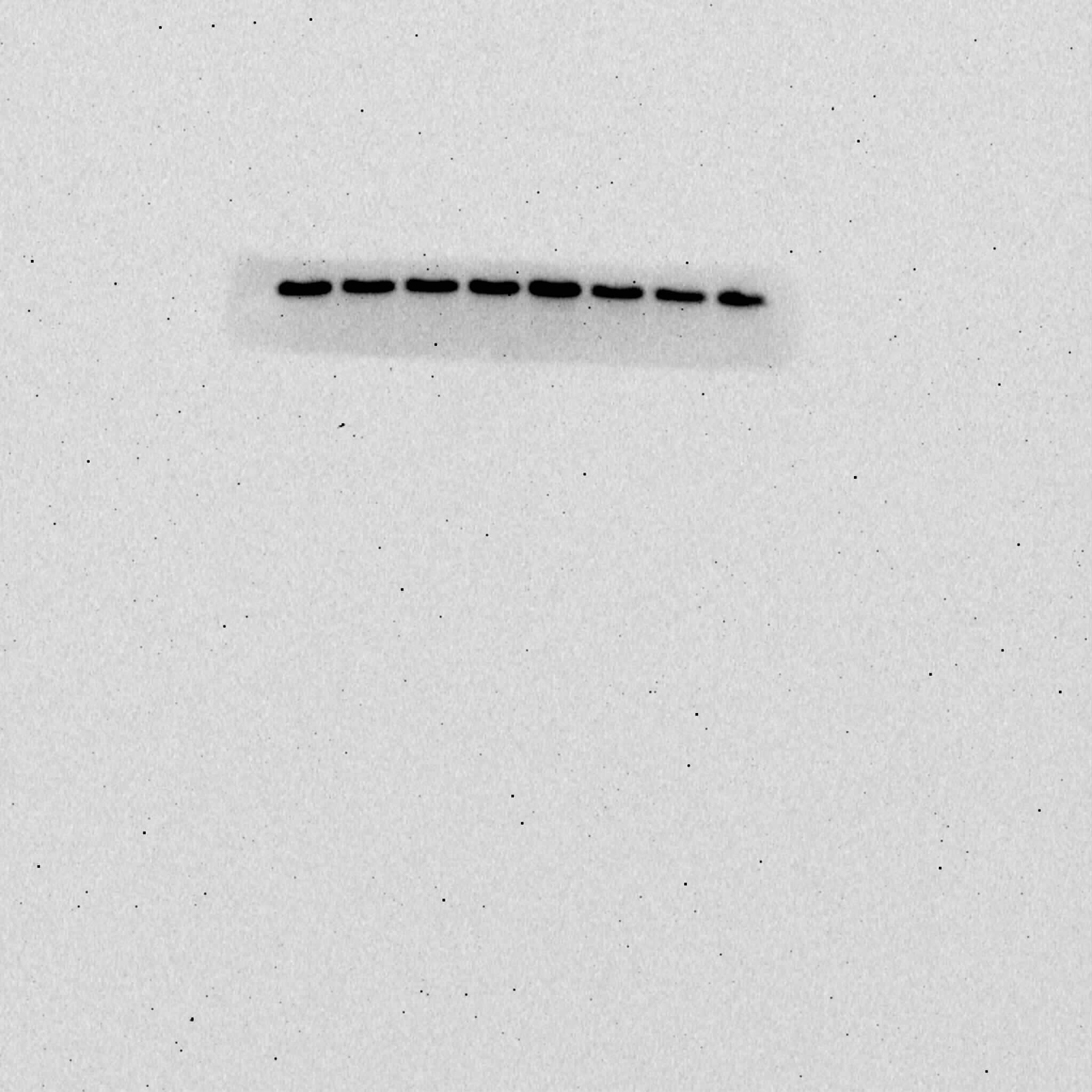

Supplement: Supplemental Information 1 [file peerj-05-3172-s002.zip › Figures 3 and 6/Fig 6/Fig6.GAPDH.jpg]
